# Elimusertib enhances cytotoxic effects of conventional chemotherapy and sensitizes to radiation in preclinical Ewing sarcoma models

**DOI:** 10.1038/s41598-026-41751-5

**Published:** 2026-03-27

**Authors:** Leonhard Koch, Maximilian Kerkhoff, Maximilian Bretschneider, Samet Dayi, Pauline Plaumann, Bahareh Sadeghi, Emma Maaßen, Emilia Hillesheim, Marc Kuballa, Christiane Schaefer, Daniel Rauh, Susanne Grunewald, Sebastian Bauer, Cläre von Neubeck, Uta Dirksen

**Affiliations:** 1 Department of Pediatrics III, University Hospital Essen, Essen, Germany; 2 West German Cancer Center Essen, University Hospital Essen, Essen, Germany; 3 German Cancer Consortium (DKTK), National Center for Tumordiseases (NCT) West, Partner Site Essen, Essen, Germany; 4 Department of Chemistry and Chemical Biology, TU Dortmund, Dortmund, Germany; 5 Department of Particle Therapy, University Hospital Essen, Essen, Germany; 6 Nordwest Krankenhaus Frankfurt, Frankfurt, Germany

**Keywords:** Cancer, Drug discovery, Oncology

## Abstract

Ewing Sarcoma (EwS) is a highly aggressive malignancy predominantly affecting young individuals, with poor prognosis in metastatic or relapsed cases. This study investigates the therapeutic potential of ataxia telangiectasia and Rad3-related protein (ATR) inhibition using the selective small-molecule inhibitor elimusertib, both as a monotherapy and in combination with cytotoxic drugs or radiotherapy. Elimusertib significantly inhibited cell proliferation and induced apoptosis in EwS cell lines. In a chorioallantoic membrane (CAM) model, elimusertib suppressed tumor initiation and reduced tumor volume. Notably, elimusertib exhibited synergistic effects with standard chemotherapeutics and radiation, enhancing antitumor efficacy. These preclinical findings suggest that ATR inhibition by elimusertib may enhance current EwS therapies and enable dose reduction of cytotoxic drugs. Further clinical evaluation is warranted to validate these findings and explore elimusertib’s translational potential in EwS treatment.

**Supplementary Information:**

The online version contains supplementary material available at 10.1038/s41598-026-41751-5.

## Introduction

Ewing sarcoma (EwS) is a highly aggressive bone and soft tissue cancer that primarily affects children, adolescents, and young adults. Despite advances in multimodal therapies including surgery, chemotherapy, and radiotherapy the prognosis for patients with metastatic or recurrent EwS remains extremely poor. While the 5-year survival rate for patients with localized disease approaches 70%, it drops to approximately 30% for those with metastatic disease. Around 15% of EwS patients present with metastases at diagnosis, highlighting the urgent need for more effective therapies^1^. Current treatment protocols typically involve polychemotherapy followed by local therapy, such as surgery and/or radiotherapy. Commonly used chemotherapeutic agents include vincristine, doxorubicin, ifosfamide, etoposide, and cyclophosphamide, which aim to reduce tumor burden and manage systemic disease^2^. However, conventional chemotherapy is associated with substantial toxicity to normal tissue, long-term health risks, and an elevated risk of secondary malignancies^3–9^. Local control of the primary tumor is critical; surgery is the mainstay when feasible and is often supplemented by radiotherapy to achieve complete tumor eradication or prevent recurrence^1,10,11^. For patients with metastatic or relapsed disease, therapeutic options remain limited and largely ineffective^1,12,13^. A hallmark of EwS is the presence of the EWSR1:FLI1 fusion protein that drives oncogenesis and promotes cell proliferation, which induces replication stress^1,14–16^, triggering DNA damage response (DDR), which is primarily regulated by three kinases: ataxia-telangiectasia mutated (ATM), ataxia telangiectasia and Rad3-related protein (ATR), and DNA-dependent protein kinase (DNA-PK). While ATM and DNA-PK primarily respond to double-strand DNA breaks, ATR responds to replication stress involving single-stranded DNA^17–19^. Due to their high level of replication stress, EwS cells are thought to be particularly reliant on ATR signaling for survival, making ATR inhibition a promising therapeutic strategy^20,21^. To systematically evaluate potential targeted therapies, we participated in the FORTRESS experiment, an international drug-screening effort using diverse sarcoma cell lines. In this context, we analyzed six novel agents in six EwS cell lines: elimusertib (ATR inhibitor), niraparib (PARP inhibitor), molibresib (BET inhibitor), borussertib (AKT inhibitor), AZD1390 (ATM inhibitor), and AZD7648 (DNA-PK inhibitor). Among these, elimusertib demonstrated the greatest efficacy based on half-maximal inhibitory concentrations (IC₅₀) values and was selected for further preclinical evaluation. This study investigates elimusertib´s potential as a therapeutic agent for EwS, both as monotherapy and in combination with standard chemotherapeutic regimens and radiation. Our findings suggest that ATR inhibition represents a promising strategy to improve therapeutic outcomes in EwS.

## Materials and methods

### Cell culture

The human EwS cell lines TC-32 and TC-71 were obtained from the cell line bank at Children’s Hospital Los Angeles, CA, USA. The CADO-ES-1 and MHH-ES-1 EwS cell lines were originally sourced from the German Collection of Microorganisms and Cell Cultures (DSMZ, Braunschweig, Germany). The STA-ET-1 and EW-7 EwS cell lines were provided by Prof. Dr. Heinrich Kovar (Children’s Cancer Research Institute, Vienna, Austria). The non-malignant human mesenchymal stem cell line G008 was kindly provided by our colleagues from the AML laboratory, Department of Pediatrics III, University Hospital Essen, Germany, by whom the cell line was established and previously described^22^. All tumor-derived cell lines were maintained in Roswell Park Memorial Institute (RPMI-1640) medium (Gibco, Thermo Fisher Scientific, Waltham, MA, USA) supplemented with 10% Fetal Bovine Serum (FBS; Biochrom GmbH, Berlin, Germany) at 37 °C in 5% CO_2_ humidified atmosphere. Cells were cultured in collagen-coated tissue culture flasks (CADO-ES-1, TC-32, TC-71) or uncoated flasks (all other cell lines). Subculturing was performed twice weekly by washing the monolayer with phosphate-buffered saline (PBS; Gibco) followed by detachment with 0.05% Trypsin-EDTA (Gibco) and resuspension in fresh RPMI medium. All cell lines were regularly tested for mycoplasma contamination using the PCR Mycoplasma Detection Kit (AppliChem GmbH, Darmstadt, Germany) in accordance with the manufacturer´s protocol.

### Compounds and reagents

Elimusertib (BAY1895344), AZ-20, berzosertib (VE-822), and ceralasertib (AZD6738) were obtained from Selleck Chemicals (Houston, TX, USA). Niraparib, molibresib, borussertib, AZD7648, and AZD1390 were kindly provided by Prof. Dr. Sebastian Bauer. Vincristine, ifosfamide, and etoposide were purchased from Cayman Chemical (Ann Arbor, MI, USA) via Biomol (Hamburg, Germany) and doxorubicin was purchased from Sigma-Aldrich (St. Louis, MO, USA). All compounds were dissolved in dimethyl sulfoxide (DMSO; AppliChem) to prepare stock solutions at 10 mM, except vincristine, which was prepared at 5 mM. Stock solutions were stored at − 20 °C. Working solutions were freshly prepared by diluting stocks in the appropriate culture medium. DMSO-only treatments were used as vehicle controls to account for solvent-related effects.

### Cell viability assay

Cell viability was assessed using either the MTT assay (3-(4,5-dimethylthiazol-2-yl)−2,5-diphenyltetrazolium bromide; Sigma-Aldrich) or the MTS assay (3-(4,5-dimethylthiazol-2-yl)−5-(3-carboxymethoxyphenyl)−2-(4-sulfophenyl)−2 H-tetrazolium; CellTiter 96^®^ AQueous One Solution Cell Proliferation Assay, Promega). Cells were detached at 70 to 80% confluence, counted using Trypan Blue exclusion (Thermo Fisher Scientific), and seeded into 96-well plates at densities optimized based on the doubling time of each cell line. After 24 h of adherence, cells were treated with inhibitors diluted in culture medium at various concentrations and incubated for 72 h. For MTT assays, 10 µL of MTT solution (5 mg/mL in PBS) was added to each well, yielding a final concentration of 0.5 mg/mL. Plates were incubated for 2 h at 37 °C, followed by addition of 100 µL isopropanol to solubilize the resulting formazan crystals. For MTS assays, 15 µL of MTS reagent was added after 72 h of drug treatment, and plates were incubated for 2 h at 37 °C. Absorbance was measured using a GENios microplate reader (Tecan, Männedorf, Switzerland) at 570 nm for MTT and 490 nm for MTS. Each experiment was performed in technical triplicate and independently repeated at least three times.

### Flow cytometry assay

To assess cell death following treatment with specific inhibitors, 1–3 × 10⁵ cells were seeded per well in 6-well plates and allowed to adhere for 24 h. Cells were then treated with inhibitors or vehicle control (DMSO) in culture medium and incubated for 48–72 h, depending on the experimental setup. Apoptosis was evaluated using the FITC Annexin V Apoptosis Detection Kit I (BD Biosciences, Franklin Lakes, NJ, USA), following the manufacturer’s instructions. This assay discriminates between early apoptotic, late apoptotic, and necrotic cell populations. Stained cells were analyzed on a BD Celesta flow cytometer (BD Biosciences). Data acquisition and analysis were performed using Kaluza 2.1 software (Beckman Coulter). Results are presented as mean ± standard deviation (SD).

### Western blot

For protein extraction, treated and untreated cell populations were trypsinized, washed with PBS, and lysed as previously described^23,24^. Protein expression was analyzed by western blot using chemiluminescence detection and visualized with a LAS-3000 Imaging System (Fujifilm, Tokyo, Japan). Primary antibodies included phospho-CHK1 (pCHK1; #2348; 1:1000), ATR (#2790; 1:1000), and cleaved PARP-1 (#9541; 1:1000), all obtained from Cell Signaling Technology (Danvers, MA, USA). beta-actin (#sc-47778; 1:1000; Santa Cruz Biotechnology, Santa Cruz, CA, USA) was used as a loading control. All primary antibodies were incubated overnight at 4 °C. Horseradish peroxidase-conjugated secondary antibodies included anti-mouse IgG (#7076; Cell Signaling Technology) and anti-rabbit IgG (#554021; BD Pharmingen), both applied at a dilution of 1:3000.

### Colony Formation Assay (CFA)

To evaluate the long-term effects of radiation and inhibitor treatment on clonogenic survival, a modified spheroidal colony formation assay was performed. EwS cells were seeded into 6-well plates at densities ranging from 3 × 10³ to 1.5 × 10⁴ cells per well, depending on the cell line, and allowed to adhere for 24 h. One hour prior to irradiation, cells were treated with elimusertib (40 nM, approximating the average IC₅₀ from MTT assays) or vehicle (DMSO) and incubated for 72 h. Following treatment, cells were embedded in a semi-solid top layer consisting of 70% RPMI, 20% fetal bovine serum (FBS), 1% penicillin/streptomycin (P/S), and 0.4% soft agar (Biozym), then reseeded into 6-well plates containing a bottom layer composed of 70% RPMI, 20% FBS, and 0.6% soft agar (Biozym). Spheroidal colonies were allowed to form over 14 days. To visualize colonies, 150 µL of MTT solution was added to each well and incubated for 2 h at 37 °C. Plates were scanned using the Epson Perfection V850 Pro scanner (Epson, Suwa, Nagano, Japan), and colonies with an area of ≥ 20 pixels were quantified using ImageJ software (NIH, Bethesda, MD, USA). Colony counts were normalized to untreated controls (DMSO) and are presented as mean ± SD for clarity.

### Chorioallantoic Membrane (CAM) Assay

The CAM assay was performed as previously described^25,26^. Briefly, fertilized chicken eggs were incubated at 37.8 °C and 60% relative humidity, with 12 automatic rotations per day. On embryonic development day 2 (EDD2), a 1.5–2 cm diameter hole was carefully cut into the eggshell and sealed with medical silk tape (Durapore™, 3 M, St. Paul, MN, USA). On EDD9, the seal was removed, and the CAM was gently perforated using a scalpel. A total of 100 µL of cell suspension (1 × 10⁶ cells resuspended in 100 µL Matrigel; Corning Inc., Corning, NY, USA) was applied directly onto the CAM surface. Tumor xenografts were allowed to develop until EDD16. At EDD16, tumors were excised, counted, and measured in three dimensions (length, width, and height). Tumor volume was calculated using the ellipsoid formula V = (4/3)πabc where a, b and c are the lengths of the three semi-axes of the ellipsoid. Experiments using the chick chorio-allantoic membrane (CAM) model do not require formal ethics approval under German and EU animal welfare regulations, as the chick embryo is not considered a protected animal before hatching. This is in accordance with previously published CAM-based studies^27,28^.

### Synergy test

To evaluate the combinatorial effects of elimusertib with standard-of-care chemotherapeutics (VIDE: vincristine, ifosfamide, doxorubicin, etoposide), EwS cells were treated with each agent as a single compound and in combination with elimusertib. Drug combination assays were performed using a 7 × 7 concentration matrix. For each drug, concentration ranges were chosen so that the respective IC₅₀ values were positioned near the center of the matrix, ensuring symmetric coverage of sub- and supra-IC₅₀ concentrations. All drug dispensing steps were performed manually using calibrated pipettes. After 72 h of treatment, cell viability was assessed using the MTS assay as described above. Absorbance values were uploaded to the SynergyFinder 3.0 platform (https://synergyfinder.fimm.fi), and synergy was quantified using the Zero Interaction Potency (ZIP) model in accordance with the platform’s user guide. The ZIP model compares dose-response relationships of single agents and their combinations to assess interaction effects. ZIP scores greater than 10 indicate synergy, scores between minus 10 and 10 indicate additive interactions, and scores less than minus 10 indicate antagonism. A ZIP score of zero reflects non-interaction. The overall synergy score represents the mean ZIP value across all tested concentration pairs. In addition, the most synergistic area score was calculated as the mean ZIP score of the most synergistic three by three dose window within the matrix to identify localized regions of maximal synergy^29^.

### Radiation

To assess the radiosensitizing potential of elimusertib in EwS cells, photon irradiation experiments were performed using an Isovolt 320 X-ray machine (Seifert–Pantak, East Haven, CT, USA) operated at 320 kV and 10 mA, with a 1.65 mm aluminum filter. Irradiations were conducted at the Institute for Medical Radiation Biology, University Hospital Essen. Cells were exposed to radiation doses of 1, 2, or 3 Gy, with non-irradiated cells serving as controls. The dose rate was 1 Gy/min, and cells were briefly maintained at room temperature during irradiation. After exposure, cells were returned to standard culture conditions (37 °C, 5% CO₂) and allowed to recover for 48–72 h, depending on the experimental design.

### Statistical analysis

Dose-response data are presented as mean ± SEM. All other data are presented as mean ± SD. Statistical comparisons between groups were performed using two-way analysis of variance (ANOVA). A p-value of < 0.05 was considered statistically significant. All analyses were conducted using GraphPad Prism, version 10.0 (GraphPad Software, San Diego, CA, USA).

## Results

### ATR inhibition reduces cell viability of Ewing sarcoma cell lines

**Fig. 1:**
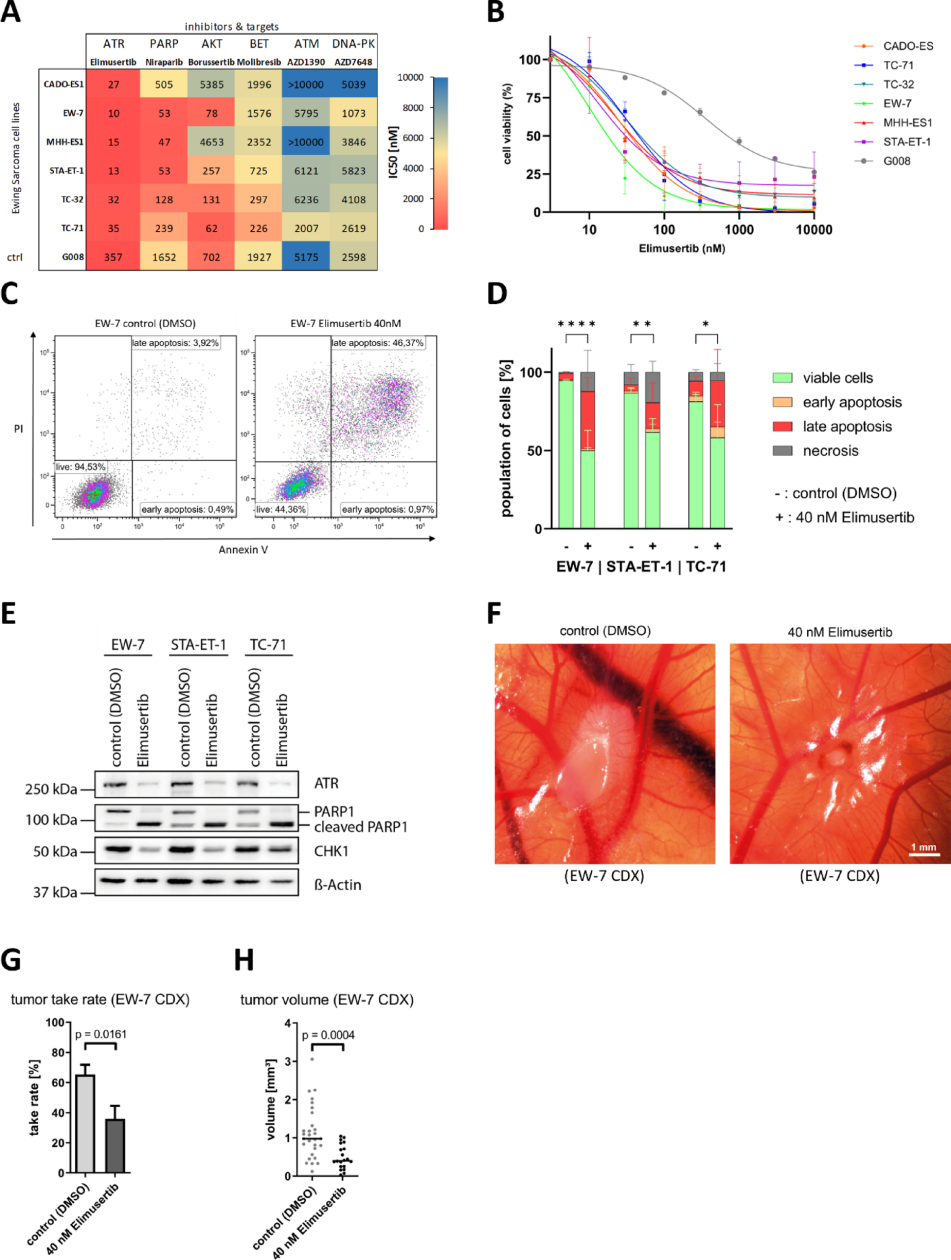
Antitumor effects of elimusertib monotherapy on EwS cells. **A** Heatmap of IC₅₀ values for six FORTRESS inhibitors in EwS cell viability assays after treatment for 72 h. G008, a non-malignant human mesenchymal stem cell line, was used as a negative control. All experiments were independently repeated at least three times. Dose-response curves and a complete table including mean IC₅₀ values ± SEM for all tested compounds are provided in the Supplementary Material. **B** Dose-response curves for elimusertib for six EwS cell lines and the non-malignant control cell line G008 after treatment for 72 h. **C** Representative dot plot from Annexin V/PI flow cytometry analysis showing apoptosis induction following treatment with 40 nM elimusertib for 72 h. **D** Quantification of Annexin V/PI flow cytometry data, indicating apoptosis levels in elimusertib-treated EwS cells. **E** Representative western blot analysis showing ATR and cleaved PARP1 protein levels in EwS cells following treatment with 40 nM elimusertib for 72 h. beta-actin was used as a loading control. Full-length blots are provided in the Supplementary Material. **F** Representative images of EW-7 cell-derived tumor xenografts on the CAM at EDD16. **G**, **H** Quantification of tumor take rate (**G**) and tumor volume (**H**) of EW-7 xenografts harvested from the CAM model. Cells were pre-treated with elimusertib before implantation. Statistical significance was determined using two-way ANOVA with Šidák’s multiple comparisons test and is indicated as follows: *p* < 0.05 (*), *p* < 0.01 (**), *p* < 0.001 (***), and *p* < 0.0001 (****).

To assess efficacy of the FORTRESS-inhibitor candidates, we performed MTT assays in multiple EwS cell lines, including CADO-ES-1, EW-7, MHH-ES-1, STA-ET-1, TC-32, and TC-71. The compounds tested included elimusertib (ATRi), niraparib (PARPi), molibresib (BETi), borussertib (AKTi), AZD1390 (ATMi), and AZD7648 (DNA-PKi). Among these, elimusertib was the most potent compound tested, with IC₅₀ values ranging from 10 nM (EW-7) to 35 nM (TC-71), followed by niraparib (IC₅₀ range: 47 nM in MHH-ES-1 to 505 nM in CADO-ES-1). The remaining inhibitors showed considerably lower efficacy in EwS cells (Fig. 1A). The non-malignant G008 cell line served as a negative control, which showed an IC₅₀ of 357 nM (Fig. 1B and Supplementary Table S1), corresponding to a significantly lower sensitivity, approximately 10-fold, compared to EwS cell lines. This indicates increased sensitivity of EwS cells compared with the non-malignant control. To benchmark elimusertib against other clinically relevant ATR inhibitors, we compared its potency to that of AZ-20, berzosertib, and ceralasertib, which are currently under investigation for various solid tumors including prostate, pancreatic, and lung cancers^30,31^. Elimusertib and berzosertib emerged as the most potent, with average IC₅₀ values of 22 nM and 40 nM, respectively, while ceralasertib and AZ-20 demonstrated reduced activity, with average IC₅₀ values of 181 nM and 103 nM. A full table with all IC_50_ concentrations can be found in Supplementary Table S1.

### ATR inhibition induces apoptosis in Ewing sarcoma cell lines

We next investigated whether elimusertib induces apoptosis in EwS cells using flow cytometry with Annexin V/PI staining. Cells negative for both markers were classified as viable; Annexin V–positive/PI–negative cells as early apoptotic; PI–positive/Annexin V–negative as necrotic; and double-positive cells as late apoptotic (representative dot plots shown in Fig. 1C). Consistent with the results of the MTT assay, treatment with 40 nM elimusertib significantly reduced the population of viable cells and increased the population of late apoptotic cells, ranging from 17% in STA-ET-1 to 37% in EW-7 (Fig. 1D). To further confirm apoptosis induction, western blot analysis revealed elevated levels of cleaved PARP1, a hallmark of programmed cell death, following elimusertib treatment (Fig. 1E).

### Inhibition of ATR reduces tumor formation potential and tumor volume in a 3D environment

To evaluate whether the effects of elimusertib observed in vitro translate into a biological three-dimensional environment, we performed a CAM assay. In this experiment, EwS cells were pre-treated with 40 nM elimusertib for 24 h and implanted onto the CAM at EDD9. This assay was designed to assess the impact of ATR inhibition on early tumor initiation and engraftment, rather than systemic therapeutic efficacy or normal-tissue tolerability. Tumors were excised and analyzed at EDD16. CAM xenografts were generated using EW-7 cells, as shown in Fig. 1F. Both the tumor take rate and tumor volumes were assessed. The take rate was significantly lower in the elimusertib-treated group (36 ± 8.6%) compared to controls (65 ± 6.4%) (*p* = 0.0161), indicating a notable reduction in tumor-forming potential (Fig. 1G). Additionally, the average tumor volume in the elimusertib group was significantly reduced, measuring 0.5 ± 0.32 mm³ versus 1.1 ± 0.68 mm³ in the control group (*p* = 0.0004; Fig. 1H).

### Combination of elimusertib with cytotoxic drugs demonstrates synergistic effects

**Fig. 2:**
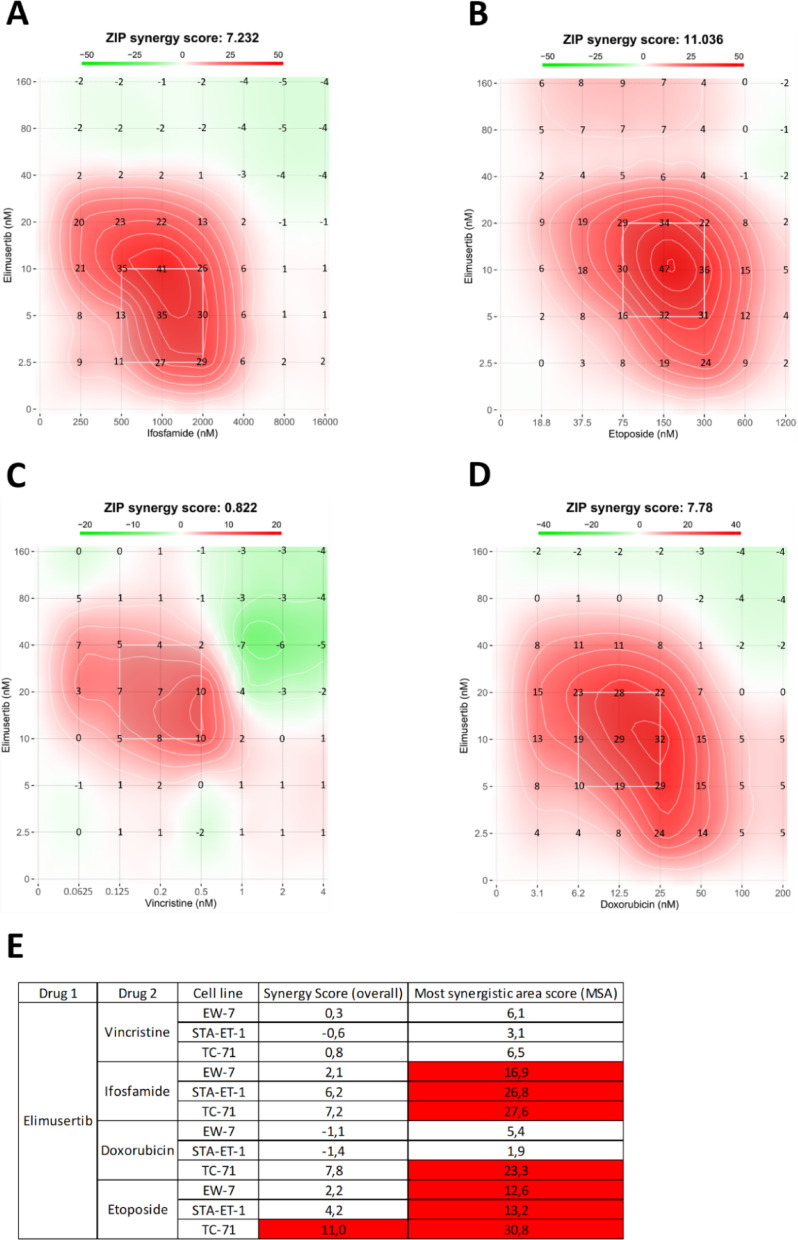
Synergistic and additive effects of elimusertib in combination with standard-of-care drugs. **A-D** Representative 2D synergy maps illustrating synergistic dose regions in red, antagonistic regions in green, and the most synergistic area (MSA; most synergistic 3 × 3 dose window (indicated by the white square)) for the combinations of elimusertib with ifosfamide (**A**), etoposide (**B**), vincristine (**C**) and doxorubicin (**D**) in TC-71. Synergy maps for EW-7 and STA-ET-1 can be found in the Supplementary Material. The value displayed above each heatmap represents the mean ZIP synergy score across the entire 7 × 7 concentration matrix. The value shown at each concentration pair represents the corresponding ZIP synergy score. **E** Table summarizing the overall synergy score (mean ZIP score across all dose combinations) and MSA scores for all drug combinations and Ewing sarcoma cell lines. Synergistic interactions, defined as ZIP scores greater than 10, are highlighted in red. Each experiment was independently repeated at least three times.

We next evaluated the combinatorial efficacy of elimusertib with standard-of-care chemotherapeutic agents—vincristine, ifosfamide, doxorubicin, and etoposide—in EwS cell lines using MTS-based viability assays. Combination treatments consistently led to a greater reduction in cell viability than either agent alone, suggesting potential synergistic effects. For elimusertib combined with ifosfamide, MSA scores ranged from 16.9 to 27.6 across EW-7, STA-ET-1, and TC-71, indicating strong synergy at low-dose combinations (Fig. 2A–D, Supplementary Fig. S4-S5). Similar synergy was observed with etoposide (MSA scores: 12.6–30.8). In contrast, combinations with vincristine produced lower MSA scores (3.1–6.5), consistent with additive effects. For doxorubicin, effects were cell line–dependent: synergy was observed in TC-71 (23.3), while STA-ET-1 (1.9) and EW-7 (5.4) showed additive responses. Notably, only mildly negative ZIP values occurred in a few high-concentration combinations. Only three individual concentration pairs showed ZIP scores slightly below − 10: EW-7: doxorubicin 80 nM + elimusertib 25 nM, EW-7: doxorubicin 80 nM + elimusertib 50 nM and STA-ET-1: doxorubicin 40 nM + elimusertib 50 nM. These values occurred at high doses where monotherapy viability was already minimal, consistent with saturation effects rather than biologically relevant antagonism (Fig. 2E).

### Combination of elimusertib with radiation demonstrates synergistic effects in apoptosis induction

**Fig. 3:**
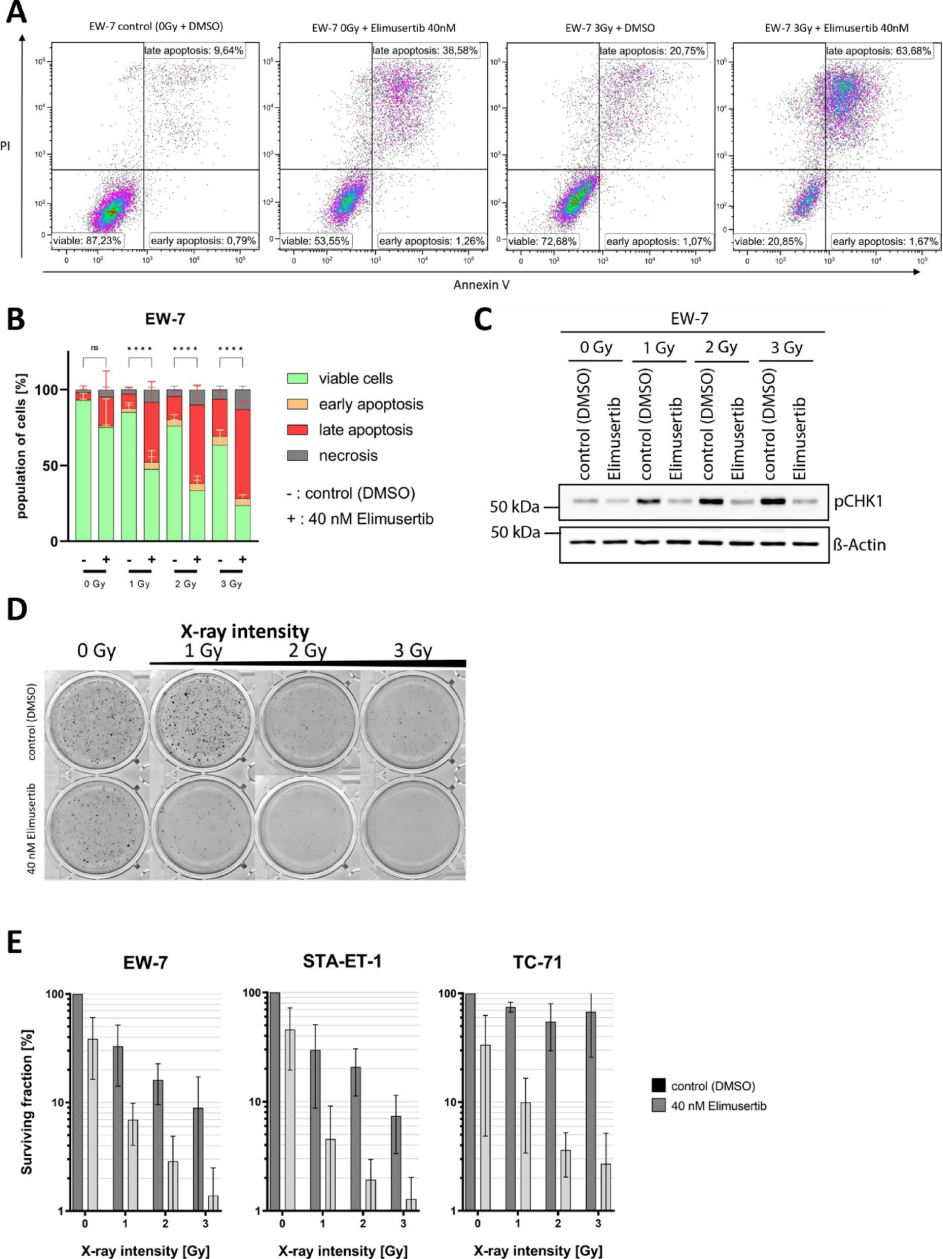
Radiosensitizing effects of elimusertib in EwS. **A** Representative dot plots from Annexin V/PI flow cytometry analysis showing apoptosis induction in EW-7 cells at increasing radiation doses (0–3 Gy), with or without 40 nM elimusertib for 48 h. **B** Quantification of Annexin V/PI FACS data, illustrating apoptosis levels across radiation doses, with or without elimusertib in EW-7. TC-71 and STA-ET-1 can be found in the Supplementary Material. **C** Representative western blot analysis showing elevated levels of p-CHK1 in cells subjected to radiation alone, and reduced levels of p-CHK1 when ATR was inhibited by elimusertib in EW-7 cells following treatment with 40 nM elimusertib for 72 h. beta-actin was used as a loading control. Full-length blots are provided in the Supplementary Material. **D** Representative images (EW-7) from a colony formation assay after radiation (1–3 Gy), with (bottom row) and without (top row) elimusertib taken after 14 days. **E** Quantification of colony formation, comparing the number of colonies following radiation alone versus radiation in combination with elimusertib. All experiments were independently repeated at least three times. Statistical significance was determined using two-way ANOVA with Tukey’s multiple comparisons test and is indicated as follows: *p* < 0.05 (*), *p* < 0.01 (**), *p* < 0.001 (***), and *p* < 0.0001 (****).

To investigate whether elimusertib enhances the apoptotic response to radiotherapy, we performed flow cytometric apoptosis assays using Annexin V/PI staining. EwS cells were pretreated with 40 nM elimusertib for 1 h and subsequently exposed to increasing doses of X-ray radiation (0, 1, 2, or 3 Gy). Induction of apoptosis was assessed 48 h post-treatment. Representative flow cytometry plots for EW-7 cells are shown in Fig. 3A. Treatment with elimusertib alone led to increased late apoptotic populations across all cell lines: 15.4% in STA-ET-1, 19.8% in TC-71, and similar elevations in EW-7, compared to control (DMSO). Radiation alone also induced apoptosis in a dose-dependent manner. At 1 Gy, late apoptosis reached 5.6% in TC-71, 7.6% in STA-ET-1, and 9.6% in EW-7. These values increased with higher doses, peaking at 8.2%, 12.6%, and 24.5%, respectively, at 3 Gy. The combination of elimusertib and radiation resulted in a significantly decreased viable cell population and a marked increase in late apoptosis in all three cell lines compared to either treatment alone. At 1 Gy, late apoptotic populations rose to 40% in EW-7 (*p* < 0.0001), 26% in STA-ET-1 (*p* = 0.0677), and 34% in TC-71 (*p* = 0.08). At 2 Gy, these values further increased to 52% in EW-7, 31% in STA-ET-1 (*p* = 0.0347), and 45% in TC-71 (*p* = 0.0078). The highest induction was observed at 3 Gy combined with elimusertib: 59% in EW-7 (*p* < 0.0001), 33% in STA-ET-1 (*p* = 0.0502), and 44% in TC-71 (*p* = 0.0096) (Fig. 3B, Supplementary Figure S6). These apoptotic responses were greater than the sum of effects observed with either treatment alone, indicating a synergistic interaction between elimusertib and radiation in EwS cells.

### Elimusertib inhibits radiation-induced checkpoint-kinase 1 phosphorylation

To determine whether elimusertib suppresses activation of the ATR–CHK1 signaling pathway following DNA damage, we measured CHK1 phosphorylation levels in EwS cells exposed to radiation with or without elimusertib. CHK1 functions downstream of ATR, and its phosphorylation is a well-established marker of ATR pathway activation in response to genotoxic stress^32^. As expected, radiation alone induced a substantial increase in phosphorylated CHK1 (p-CHK1) compared to control (DMSO), confirming the effective activation of the DNA damage response. In contrast, pretreatment with elimusertib reduced radiation-induced CHK1 phosphorylation. Cells exposed to both elimusertib and radiation exhibited much lower p-CHK1 levels compared to those treated with radiation alone (Fig. 3C). These findings indicate that elimusertib effectively inhibits ATR activity and its downstream target CHK1, thereby suppressing the radiation-induced DNA damage response.

### Elimusertib and radiation synergistically reduce colony formation

To further evaluate the potential synergistic effects of ATR inhibition and radiation on long-term survival and proliferative capacity in EwS cells, a colony formation assay (CFA) was conducted. Cells were treated with 40 nM elimusertib 1 h prior to exposure to increasing doses of X-ray radiation (0, 1, 2, and 3 Gy). Representative images (EW-7) are shown in Fig. 3D. Elimusertib treatment alone resulted in a moderate reduction in colony formation. Compared to untreated controls, the percentage of colonies formed following elimusertib monotherapy was 39 ± 22% in EW-7, 46 ± 26% in STA-ET-1, and 34 ± 29% in TC-71. Radiation alone also decreased colony numbers in a dose-dependent manner. A 1 Gy dose reduced colony formation to 33 ± 19% in EW-7, 30 ± 21% in STA-ET-1, and 75 ± 8% in TC-71. The combination of elimusertib and radiation led to a substantially greater reduction in colony formation than either treatment alone. Notably, treatment with 40 nM elimusertib combined with 1 Gy irradiation was more effective than 3 Gy radiation alone, reducing colony formation to 6.9 ± 2.9% in EW-7, 4.6 ± 4.6% in STA-ET-1, and 10 ± 6.6% in TC-71 (Fig. 3E). These findings demonstrate that the combination of elimusertib and radiation reduced colony formation more than the sum of their individual effects, indicating a synergistic interaction.

## Discussion

This study evaluated six targeted inhibitors as part of the FORTRESS initiative, a collaborative drug-screening effort focused on sarcoma models. Among the tested agents, four compounds, namely borussertib, molibresib, AZD1390, and AZD7648, failed to demonstrate efficacy at clinically relevant concentrations. In contrast, the ATR inhibitor elimusertib and the PARP inhibitor niraparib significantly reduced EwS cell viability. Although PARP inhibitors have shown promise in preclinical EwS models^33,34^, their limited clinical efficacy as monotherapies has been well documented^35^. These limitations led us to further investigate elimusertib, which demonstrated superior potency. ATR is a central regulator of the DDR, particularly in response to replication stress and single-stranded DNA lesions^36,37^. ATR activation facilitates cell cycle arrest and DNA repair, enabling tumor cells to survive genotoxic stress. EwS cells, driven by the EWSR1::FLI1 fusion oncogene, are characterized by high levels of replication stress and genomic instability. This dependency renders them particularly vulnerable to ATR inhibition^21^. Inhibiting ATR induces synthetic lethality by disabling a critical survival pathway in the presence of therapy-induced DNA damage or replication-associated lesions, thereby creating a therapeutically exploitable vulnerability^38^. While elimusertib demonstrates strong activity in Ewing sarcoma models, ATR inhibitors are known to exert potent antitumor effects across multiple cancer types; therefore, the findings presented here should not be interpreted as EwS-specific sensitivity but rather as a characterization of EwS responsiveness within the broader context of ATR-inhibitor susceptibility. Elimusertib treatment alone significantly impaired EwS cell proliferation and induced apoptosis, as confirmed by flow cytometry and PARP1 cleavage. Elimusertib reduced both tumor take rate and tumor volume in the chorioallantoic membrane model, consistent with previous findings in other tumor models^21,39,40^. Because elimusertib was applied to the cells prior to implantation, the CAM assay in this study represents a *semi-in vivo* model designed to determine whether the *in vitro* effects of ATR inhibition translate into a biological 3D environment and whether ATR blockade impairs tumor initiation. These data confirm that elimusertib exerts robust antitumor effects as a monotherapy in EwS, both in vitro and in a biologically relevant *semi-in vivo* setting. The potential of ATR inhibitors, such as elimusertib, to enhance chemotherapy efficacy is a topic of great interest across various cancer types, both *in vitro* and *in vivo*^41–47^. This strategy has been proven to be effective in several clinical trials that combined ATR inhibitors with DNA-damaging agents^48–51^. In EwS cell-derived xenografts, low-dose cisplatin has been shown to synergize with ATR inhibition^52^. However, preclinical data on combining ATR inhibitors with standard-of-care therapies in EwS were lacking. To fill this void and assess the potential risks of antagonistic interactions in a clinical trial context, we conducted a synergy experiment. We observed consistent synergy between elimusertib and low-dose ifosfamide and etoposide, with additive effects for doxorubicin and vincristine. Interestingly, TC-71 cells showed the most pronounced synergy despite being the least sensitive to elimusertib as a single agent. Only very few antagonistic interactions at high individual doses were detected, suggesting that elimusertib can be safely integrated into existing chemotherapy regimens. These effects were evident at clinically achievable doses of elimusertib (2.5–20 nM)^50,51^, highlighting its translational potential and supporting its advancement toward clinical evaluation. In relapsed or refractory disease, where outcomes with standard-of-care therapy remain poor, combining elimusertib with DNA-damaging agents may offer a rational and impactful therapeutic strategy^1^. The observed synergy is mechanistically plausible. These agents primarily induce various forms of DNA damage, including strand breaks, crosslinks, and DNA intercalation, which trigger ATR activation. Inhibiting ATR in this context blocks damage sensing and repair, thereby enhancing cytotoxicity^38^. Vincristine, in contrast, disrupts microtubule dynamics without directly damaging DNA, which may explain its additive interaction with elimusertib. This mechanistic alignment reinforces the rationale for combining ATR inhibitors with DNA-damaging agents in EwS. Radiotherapy is integral to EwS management, particularly when surgical resection is infeasible^1,11^. However, re-irradiation is often contraindicated due to toxicity, and young patients face elevated risks of long-term complications and secondary malignancies^10,53,54^. Since radiation induces DNA damage that activates ATR, inhibiting ATR is a rational strategy for radiosensitization^55^. This concept has demonstrated efficacy in other cancer types^56,57^. Our results showed that combining 40 nM elimusertib with 1 Gy of radiation exceeded the effect of 3 Gy radiation alone, reducing clonogenic survival and increasing apoptosis in a synergistic manner. These findings suggest that elimusertib may enhance the efficacy of radiotherapy and/or enable dose de-escalation, with important implications for improving safety and long-term outcomes. Multiple ATR inhibitors, including elimusertib, berzosertib, ceralasertib, and gartisertib, are currently in clinical trials for solid tumors^38,58,59^. These agents have been shown to be orally bioavailable and well tolerated, including in combination regimens^38,60,61^. Our findings add to this growing body of evidence, suggesting that ATR inhibition is not only feasible in EwS but may broaden the therapeutic index of existing treatment strategies. Future work should focus on optimizing combination dosing regimens and identifying biomarkers predictive of ATR inhibitor sensitivity. Additionally, while ATM (ataxia-telangiectasia mutated) inhibition did not yield significant monotherapeutic activity in our study, its role in radiation-induced double-strand break repair via the ATM–CHK2 pathway remains of interest^19,62,63^. Dual targeting of ATR and ATM may warrant further exploration, especially in the context of high-dose or fractionated radiation^36^. Clinical trials will be essential to validate these preclinical findings and to assess the safety and efficacy of elimusertib in patients with EwS. In summary, these findings demonstrate that elimusertib exerts potent anticancer activity in EwS models and synergizes with both DNA-damaging chemotherapeutics and radiation. Our findings support the integration of ATR inhibitors into EwS treatment protocols and provide a compelling rationale for their continued clinical development.

## Supplementary Information

Below is the link to the electronic supplementary material.

Supplementary Material 1

## Data Availability

The datasets generated and analyzed during the current study are not publicly available but are available from the corresponding authors on reasonable request.
