# Supplementary material for: Elimusertib enhances cytotoxic effects of conventional chemotherapy and sensitizes to radiation in preclinical Ewing sarcoma models
Source: Sci Rep. 2026 Mar 27;16:10953. doi: 10.1038/s41598-026-41751-5 (PMC13039535; doi:10.1038/s41598-026-41751-5)

Elimusertib enhances cytotoxic effects of conventional chemotherapy and sensitizes to radiation in preclinical Ewing sarcoma models

Leonhard Koch[1,2,3]*, Maximilian Kerkhoff[1,2,3], Maximilian Bretschneider[1,2,3], Samet Dayi[1,2,3], Pauline Plaumann[1,2,3], Bahareh Sadeghi[1,2,3], Emma Maaßen[1,2,3], Emilia Hillesheim[1,2,3], Marc Kuballa[1,2,3], Christiane Schaefer[1,2,3], Daniel Rauh[4], Susanne Grunewald[2,3], Sebastian Bauer[2,3], Cläre von Neubeck[5,6], Uta Dirksen[1,2,3]*

[1] Department of Pediatrics III, University Hospital Essen, Essen, Germany

[2] West German Cancer Center Essen, University Hospital Essen, Essen, Germany

[3] German Cancer Consortium (DKTK), National Center for Tumordiseases (NCT) west, partner site Essen, Germany

[4] Faculty of Chemistry and Chemical Biology, TU Dortmund, Dortmund, Germany

[5] Department of Particle Therapy, University Hospital Essen, Essen, Germany

[6] Nordwest Krankenhaus Frankfurt

*Corresponding Authors; leonhard.koch@uk-essen.de; uta.dirksen@uk-essen.de

SUPPLEMENTARY MATERIAL:

Supplementary Table S1: IC_{50} values (mean ± SEM) of targeted inhibitors across Ewing sarcoma cell lines and G008 control

	inhibitors & targets								
	ATR	PARP	AKT	BET	ATM	DNA-PK	ATR	ATR	ATR
cell lines	**Elimusertib**	**Niraparib**	**Borussertib**	**Molibresib**	**AZD1390**	**AZD7648**	**Berzosertib**	**Ceralasertib**	**Az-20**
**CADO-ES1**	26.7±2	505±140	5385±2339	1996±1167	13374±6441	5039±853			
**EW-7**	9.7±1.3	53±12	77.9±7.6	1576±961.7	5795±2392	1073±770.6	40.7±13.6	203±31.7	108.9±22.5
**MHH-ES1**	15.1±4.7	46.6±11.1	4653±3118	2352±1271	14422±8981	3846±2046	36.8±11.4	113.4±11.2	49.9±1.8
**STA-ET-1**	12.8±1.3	53.3±13.3	257.2±234.1	725±287	6121±3043	5823±3623			
**TC-32**	32±5.5	128.4±44.2	131.3±17.7	296.8±22.4	6236±2475	4108±625.8			
**TC-71**	34.6±2.3	239±25.6	61.7±36.5	225.8±18.3	2007±1163	2619±1223	41±16.3	228.7±62.9	149.4±25.7
**G008**	357±45	1652±1117	702±361	1927±544	5175±1265	2598±575	2176±278	6126±2466	1703±227

Supplementary Figure S2: Raw data (dose-response curves) for the table in Fig. 1A

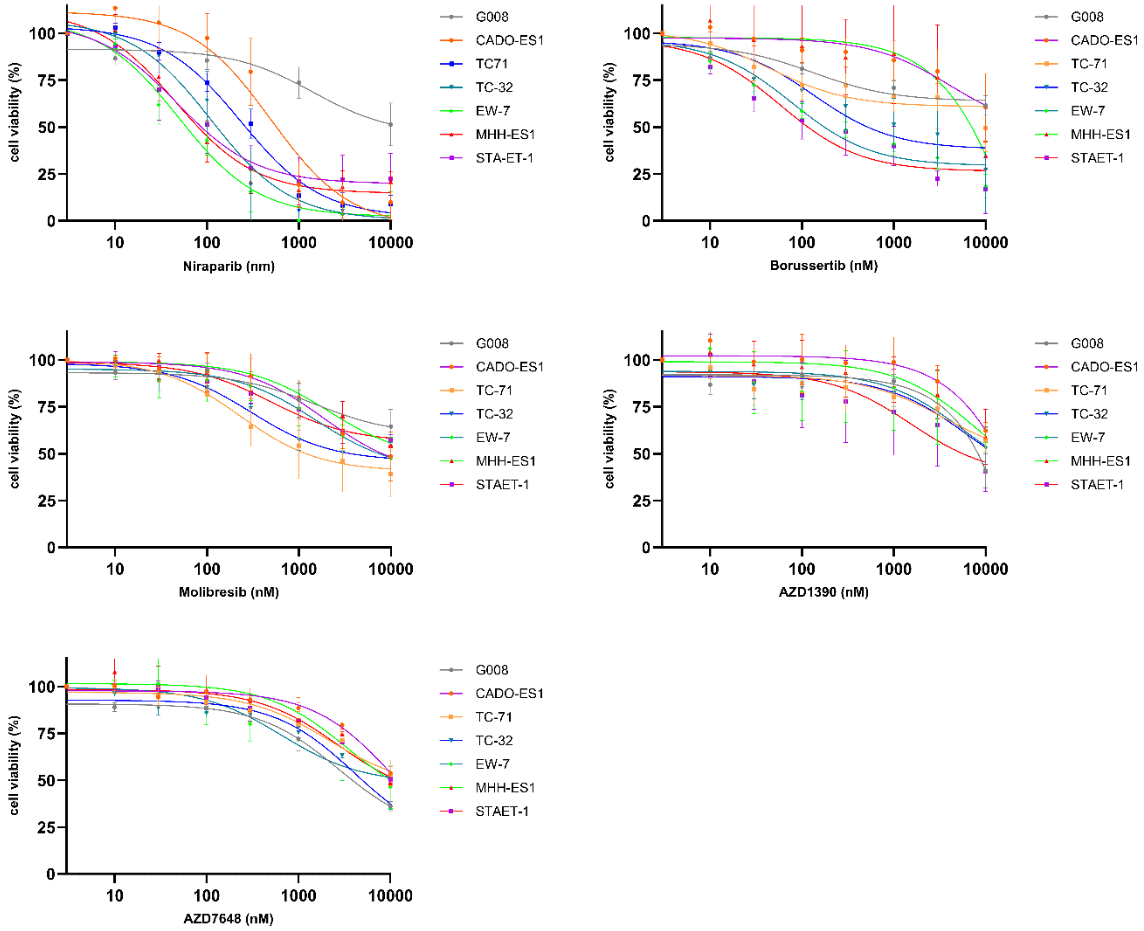

Supplementary Figure S3: Raw data (dose-response curves) for the table in Supplementary Fig. S1

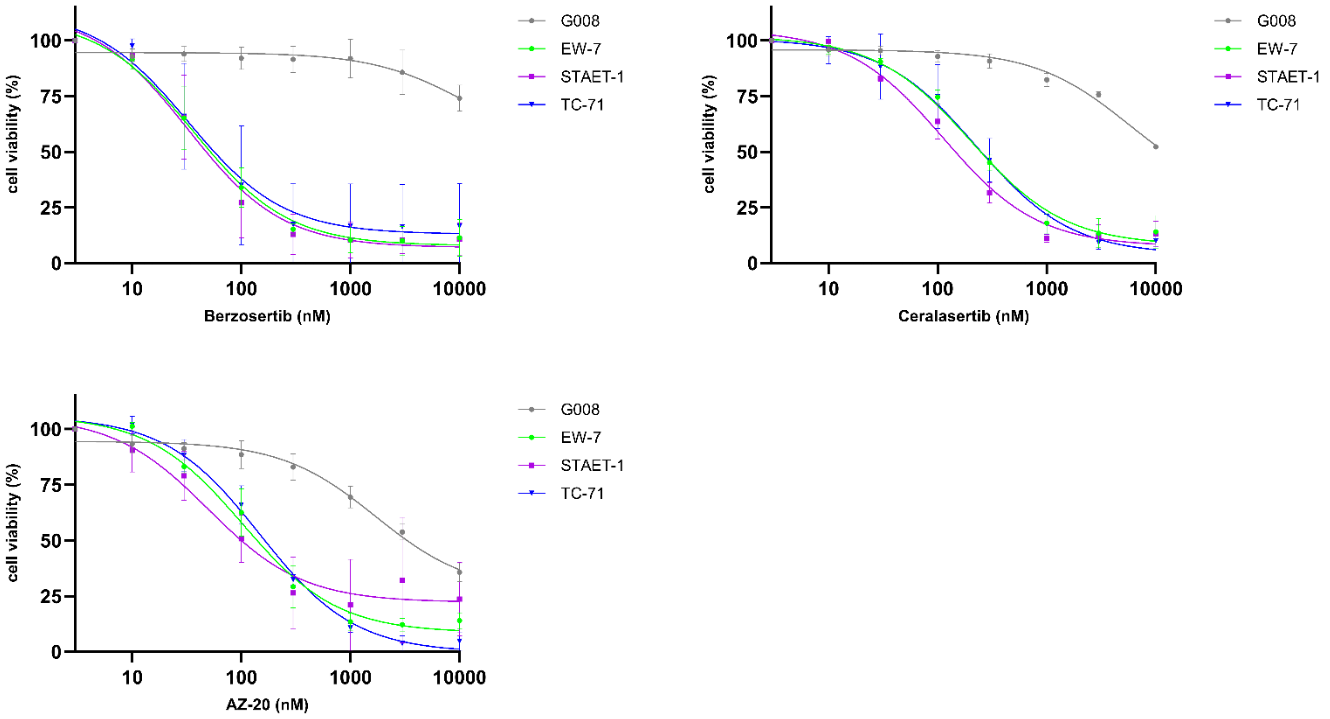

Supplementary Figure S4: Synergy maps of elimusertib in combination with SOC drugs in EW-7

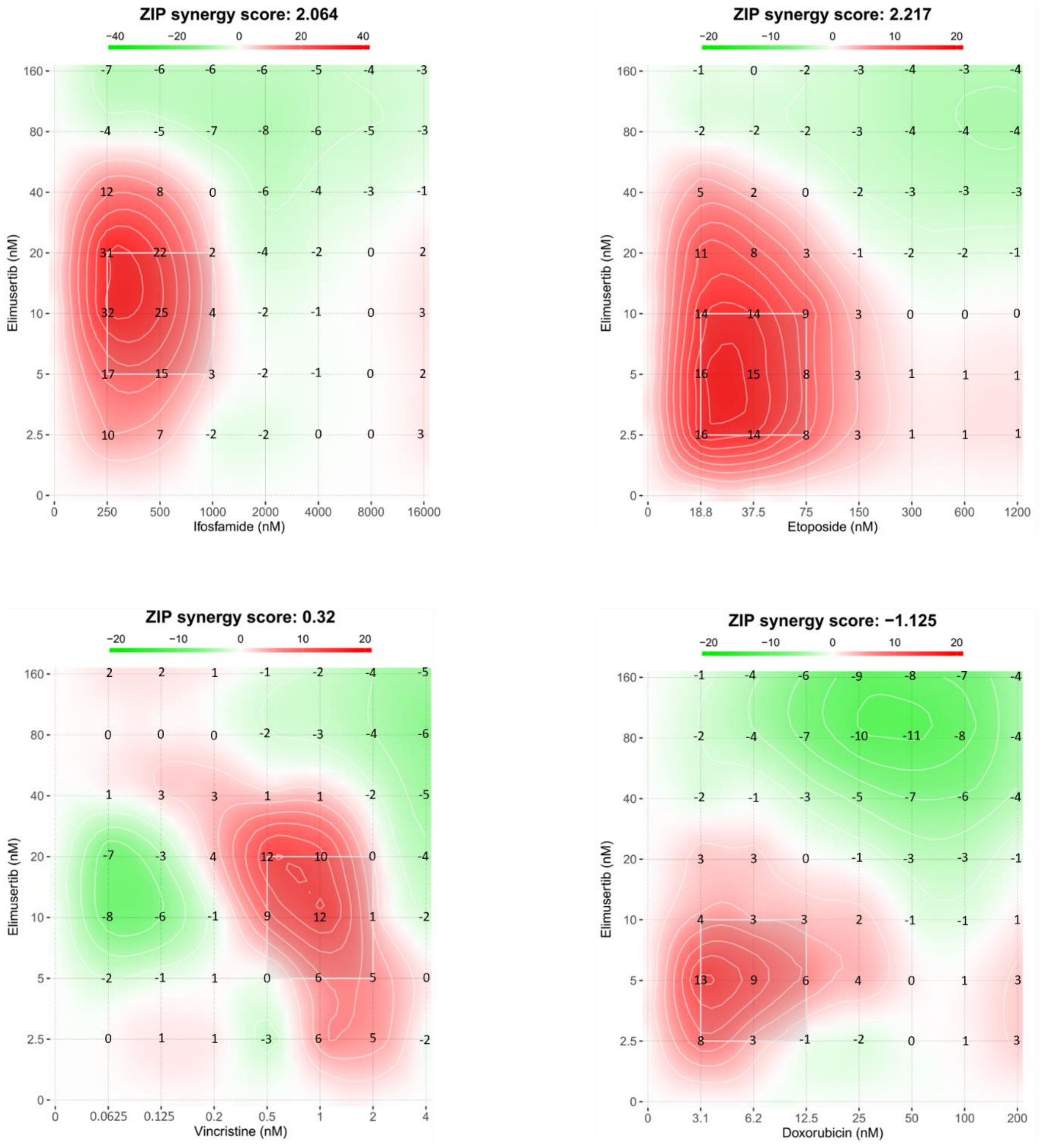

Supplementary Figure S5: Synergy map of elimusertib in combination with SOC drugs in STA-ET-1

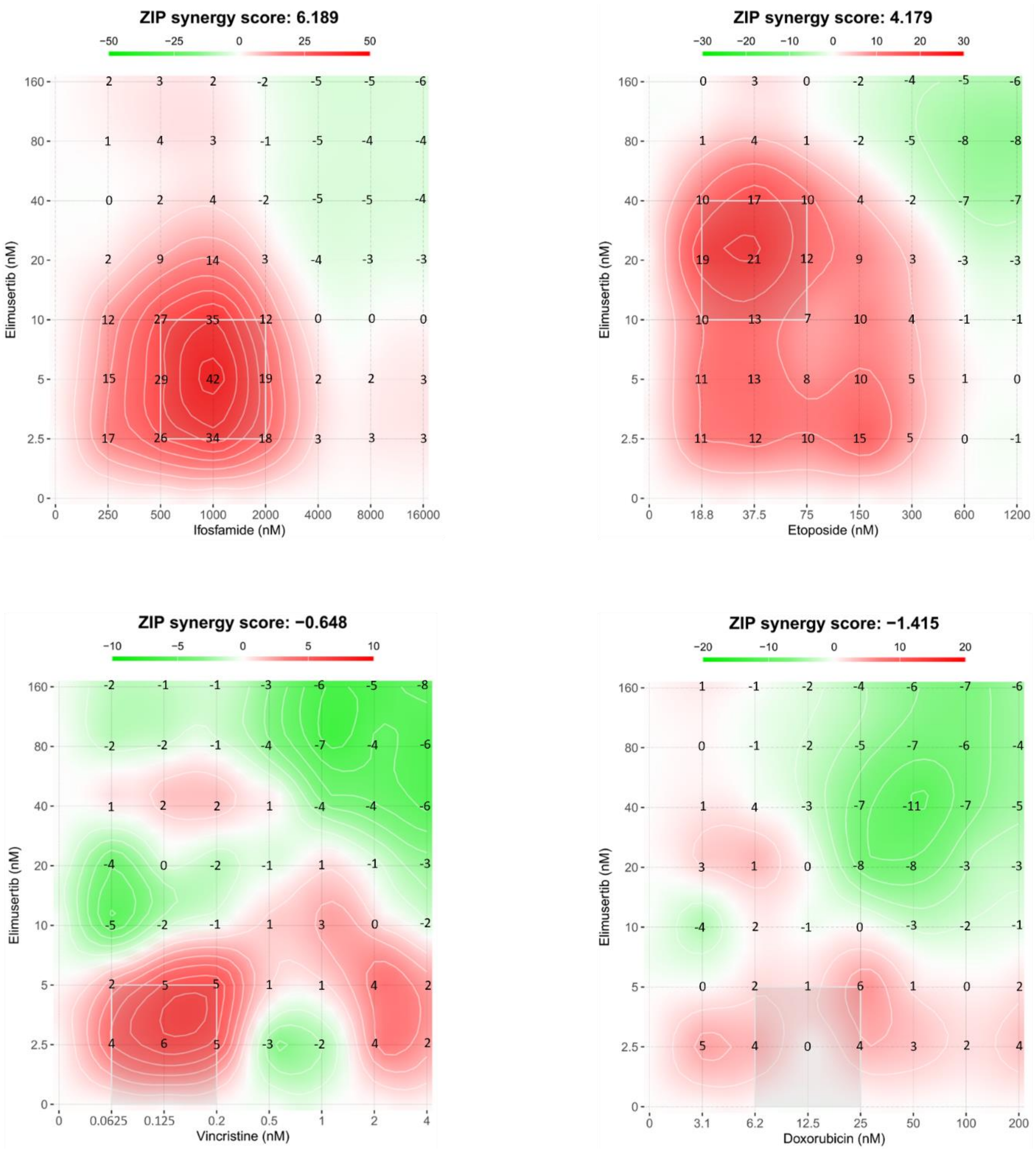

Supplementary Figure S6: Flow cytometric analysis of apoptosis in STA-ET-1 and TC-71 cells following elimusertib treatment

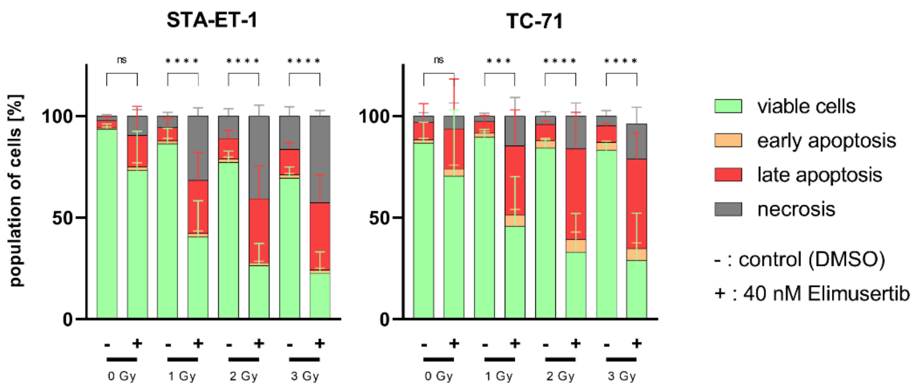

Supplementary Figure S7: Original western blot from Fig. 1E with labeling

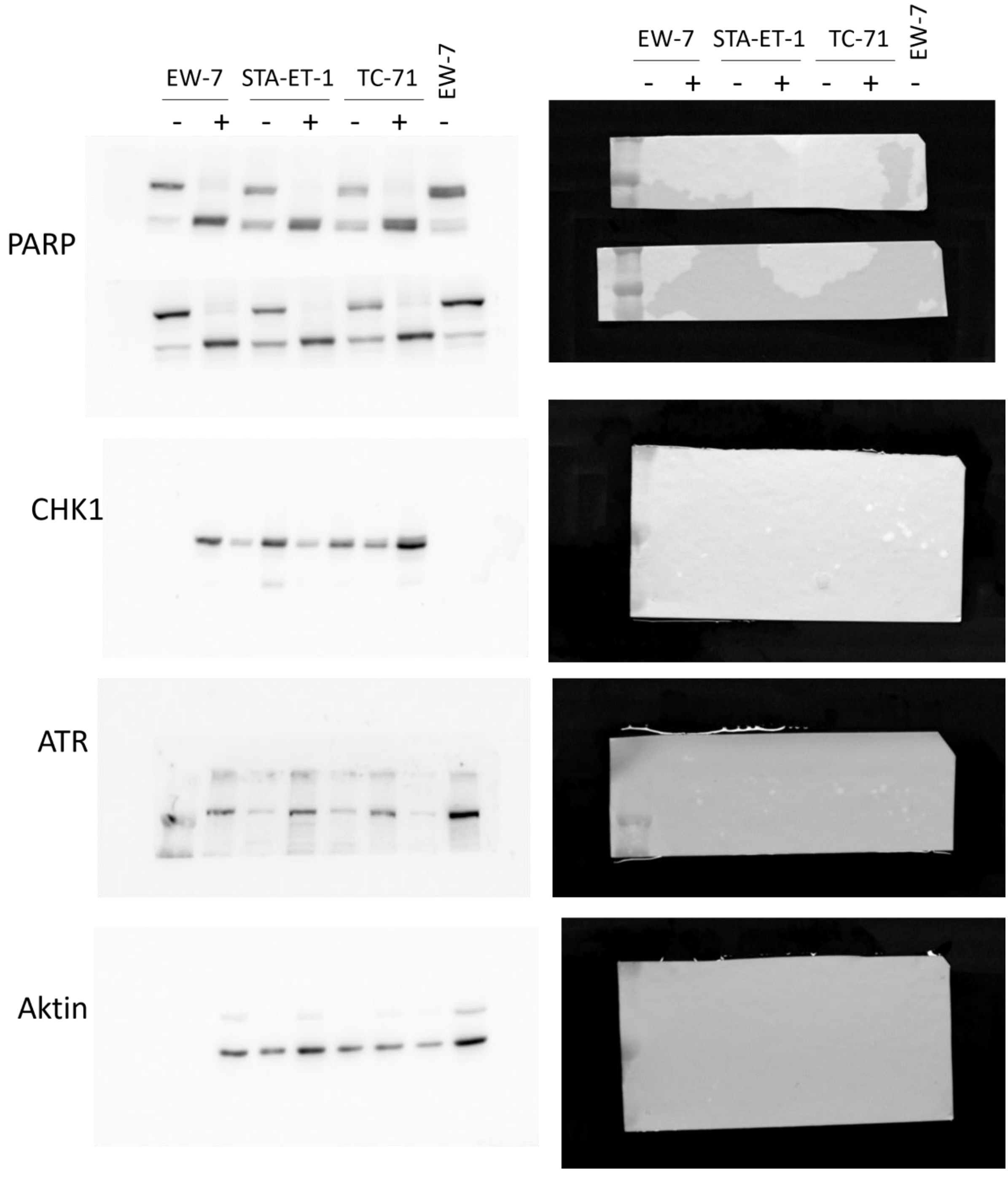

Supplementary Figure S8: Raw full images from Fig. 1E without labeling

Full image - PARP

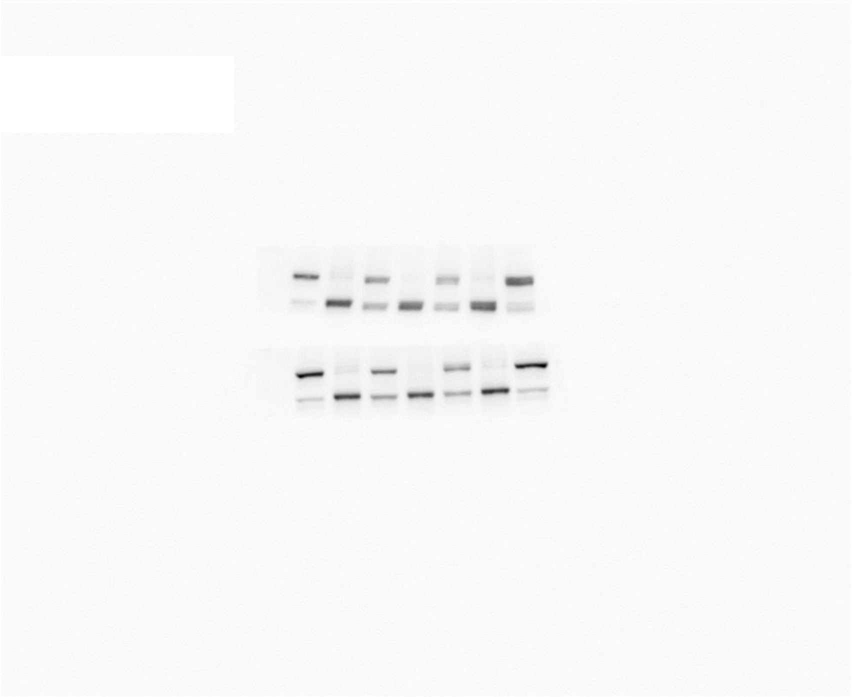

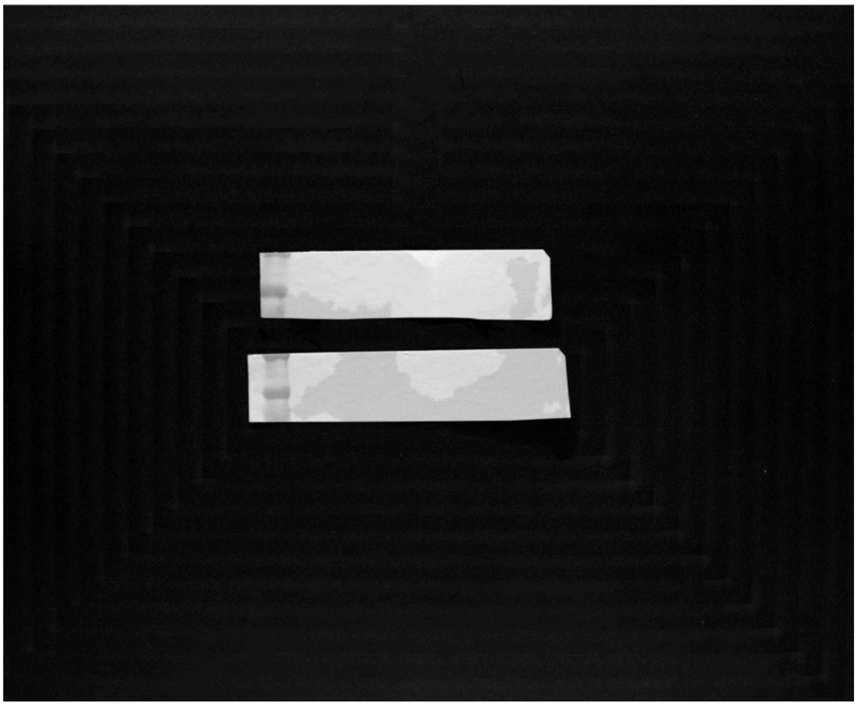

Full image - ATR

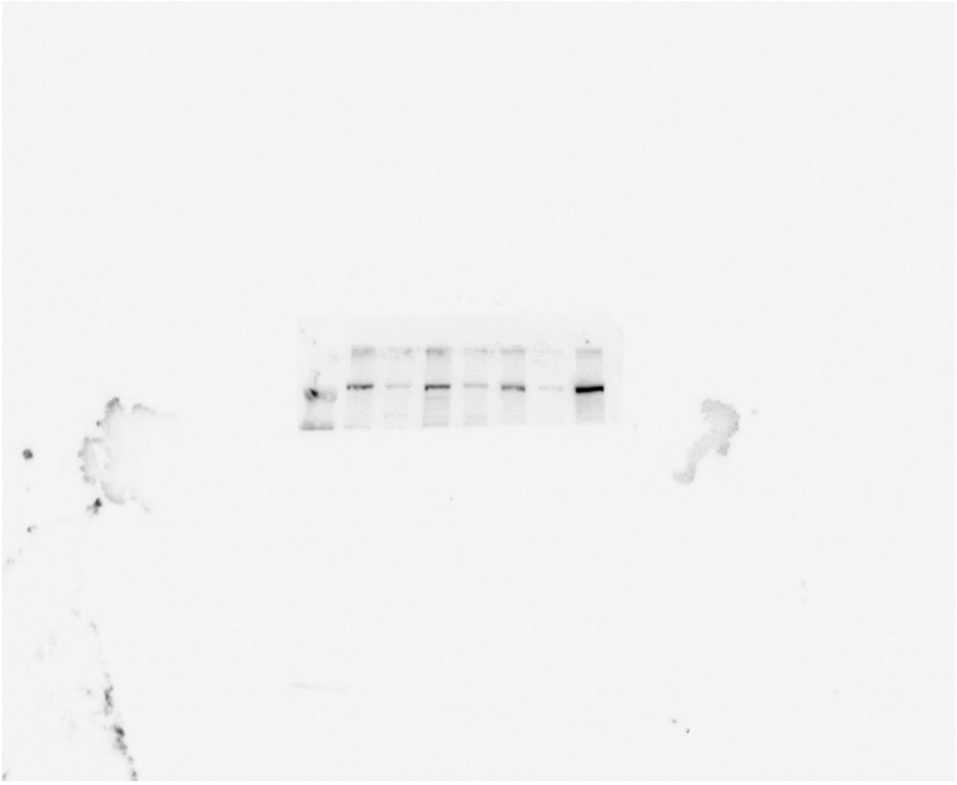

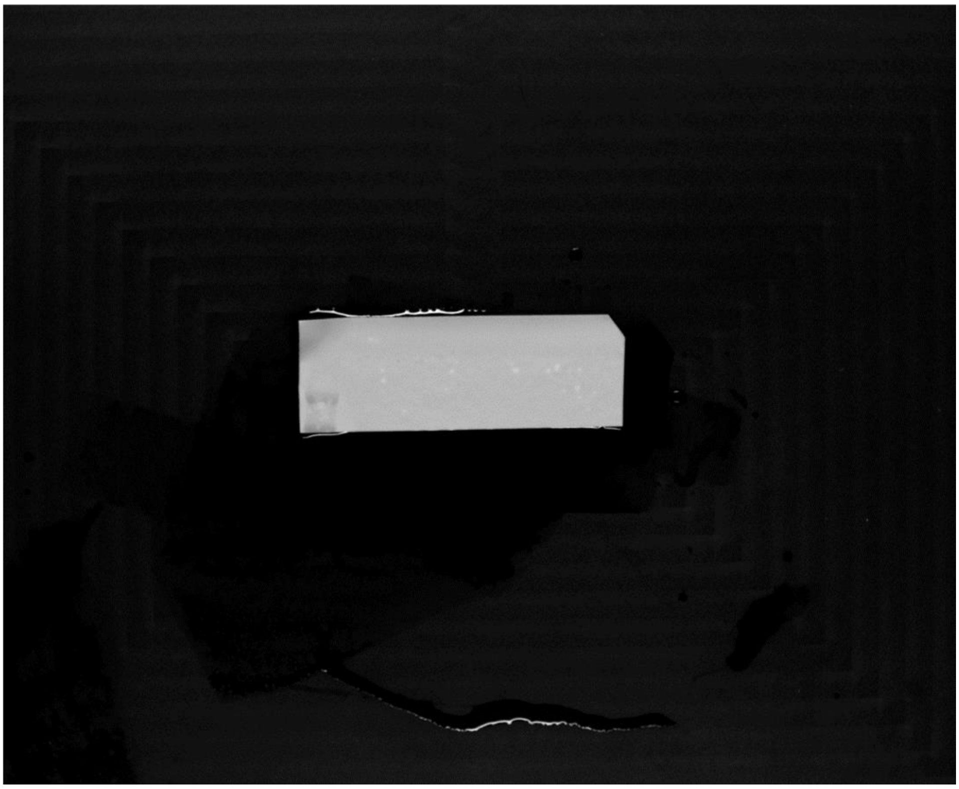

Full image – ß-Aktin

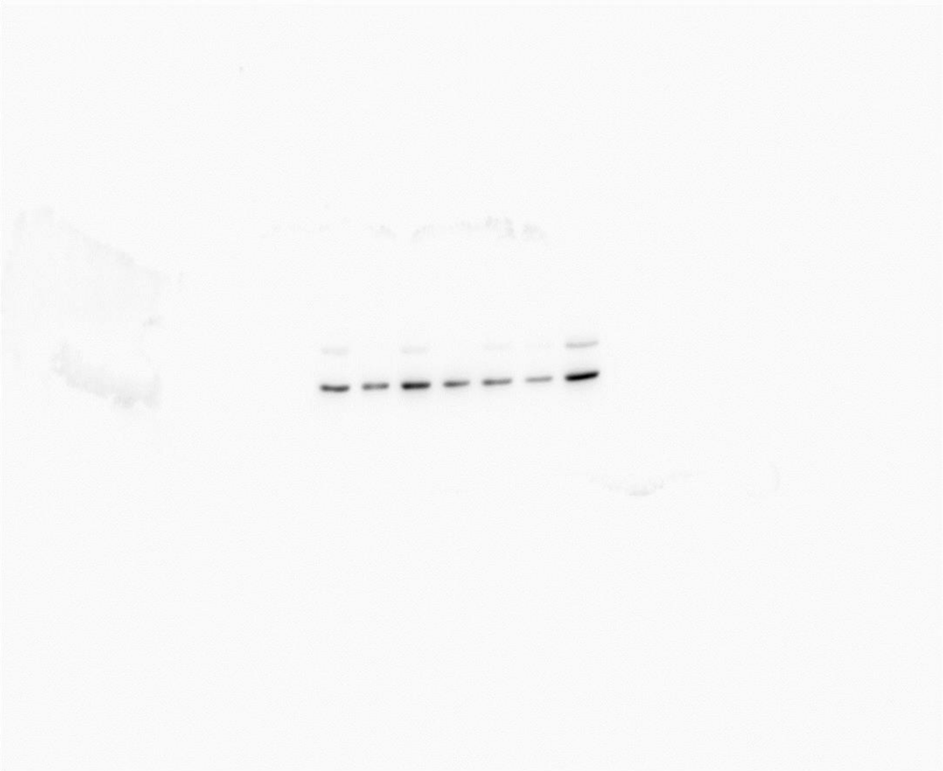

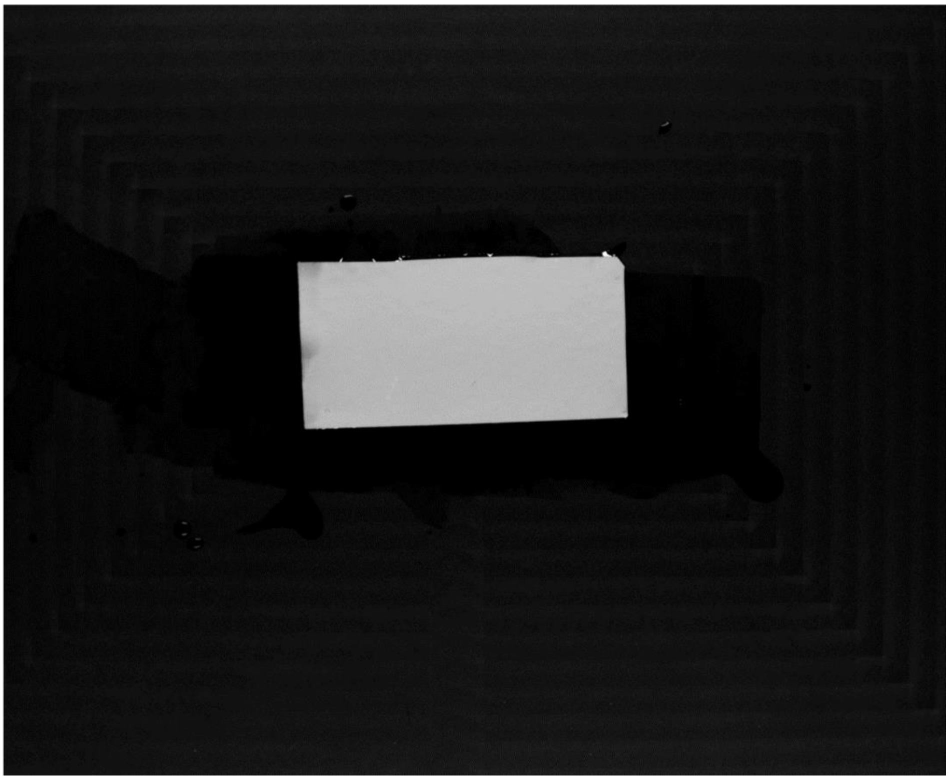

Full image – CHK1

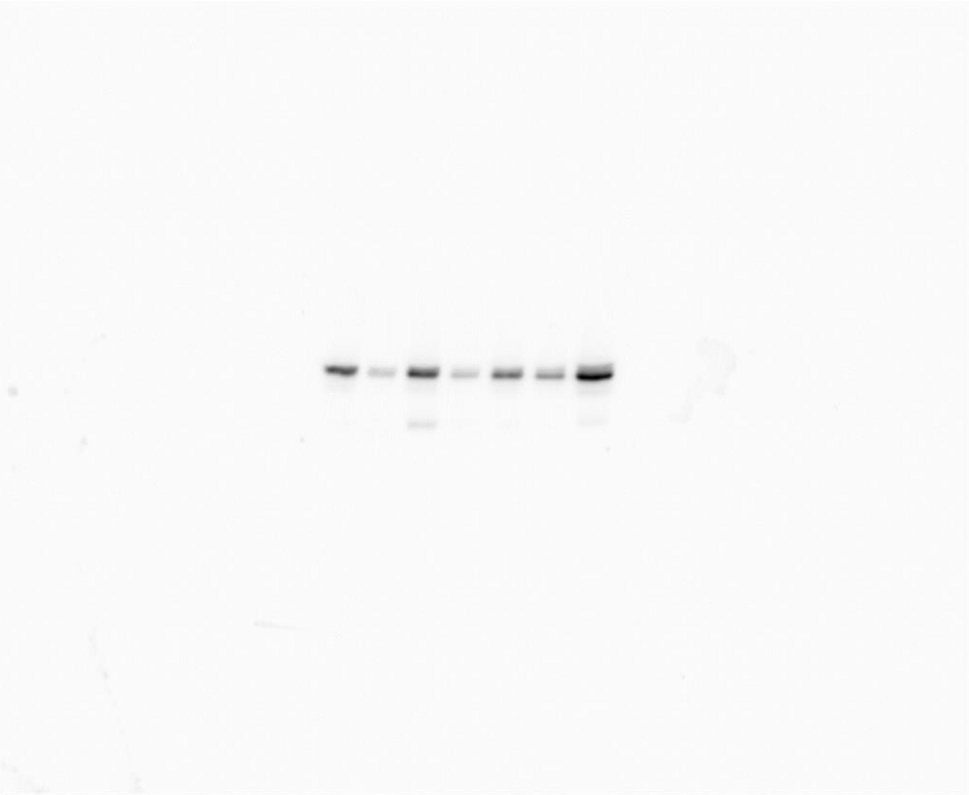

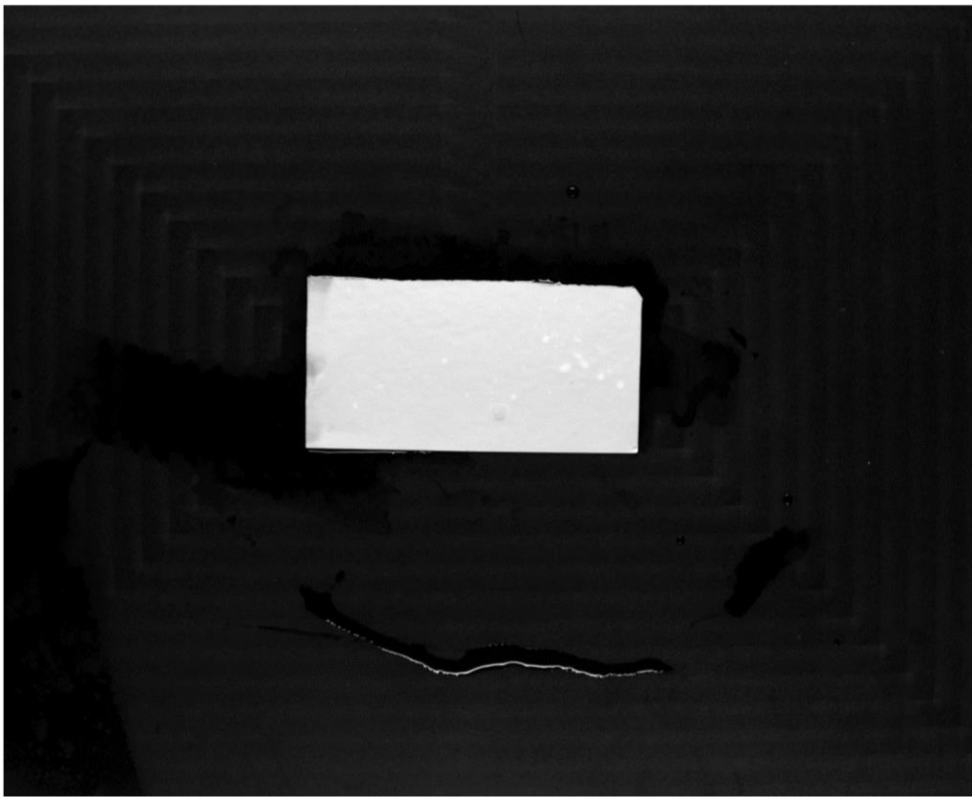

Supplementary Figure S9: Original western blots from Fig. 3C with labeling; cell line EW-7

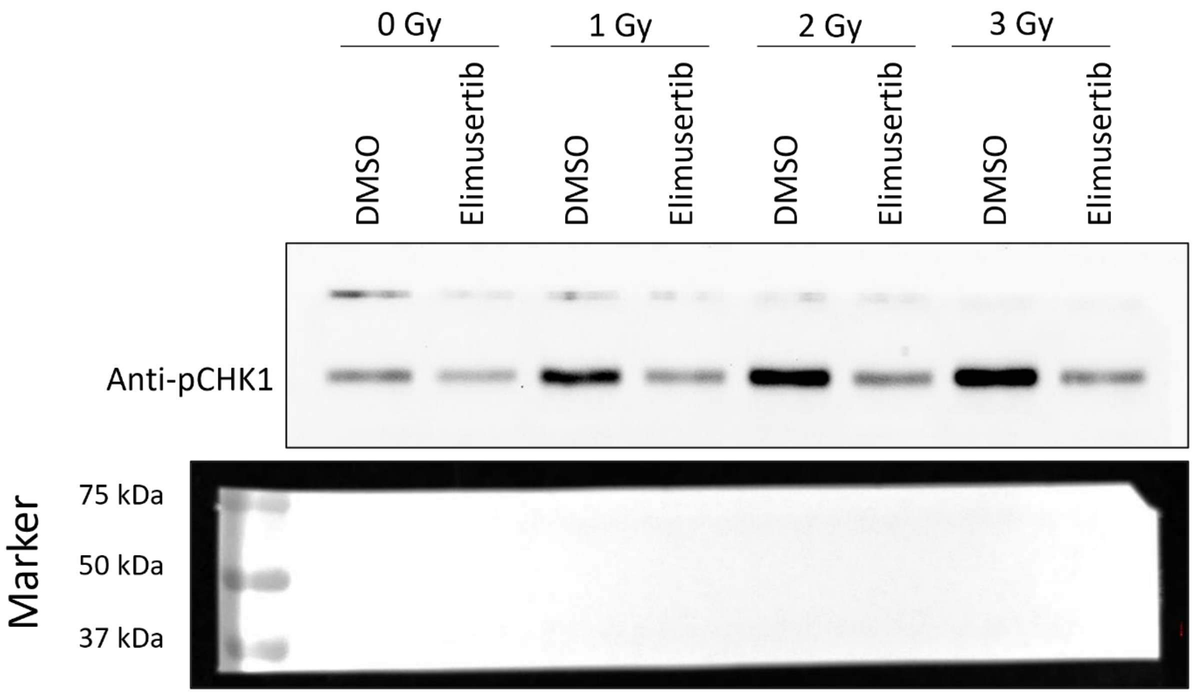

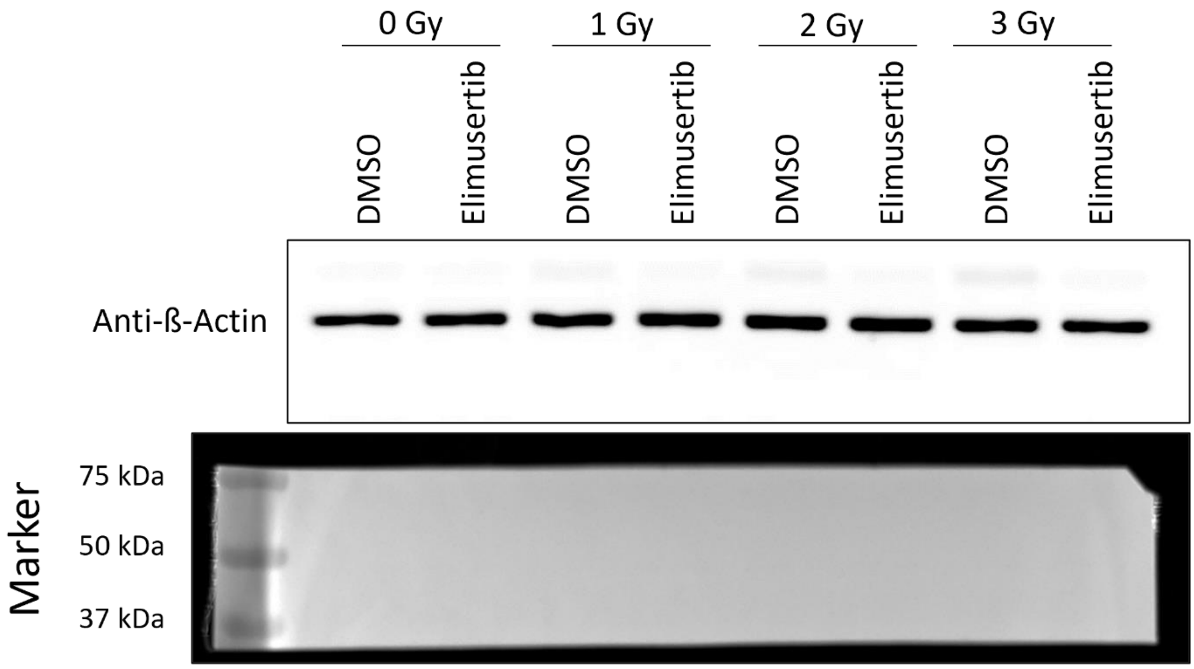

Supplementary Figure S10: Raw full images from Fig. 3C without labeling

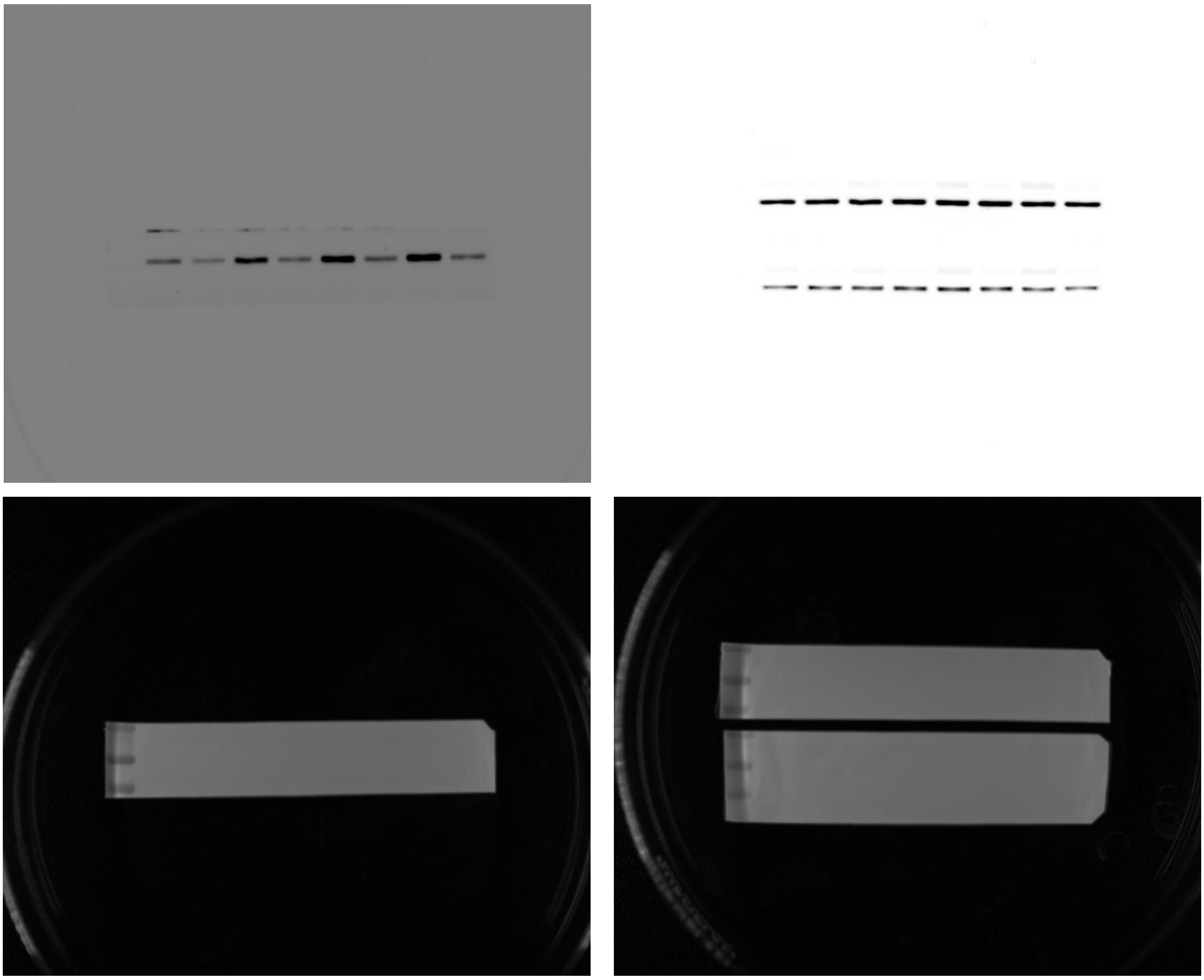

Supplement: Supplementary file 1 — Supplementary Material 1 [file 41598_2026_41751_MOESM1_ESM.pdf]
